# How to Read an EEG: A Step-by-Step Guide

**DOI:** 10.1212/NE9.0000000000200208

**Published:** 2025-03-07

**Authors:** Michael R. Butler, Kaley J. Marcinski Nascimento, Sándor Beniczky, Fábio A. Nascimento

**Affiliations:** 1 Department of Neurology, Washington University School of Medicine, St. Louis, MO; and; 2 Department of Clinical Neurophysiology, Aarhus University Hospital, Aarhus and Danish Epilepsy Centre, Dianalund, Denmark.

We present a step-by-step infographic (Figure) to aid educators in teaching EEG interpretation. First, readers should learn about settings and filters.^e1^ Second, educators should emphasize the importance of reviewing video-EEG elements and technologists' observations to determine the patient's level of consciousness. Third, the mechanics behind defining the EEG background should be discussed and demonstrated. Fourth, educators should teach trainees to recognize normal variants and artifacts.^e2^ Fifth, trainees should be instructed on identification of epileptiform and nonepileptiform interictal abnormalities^e3^ as well as ictal findings.^1,2^ Finally, educators should remind trainees to interpret extracted EEG features in light of the clinical context.^e4^

**Figure:**
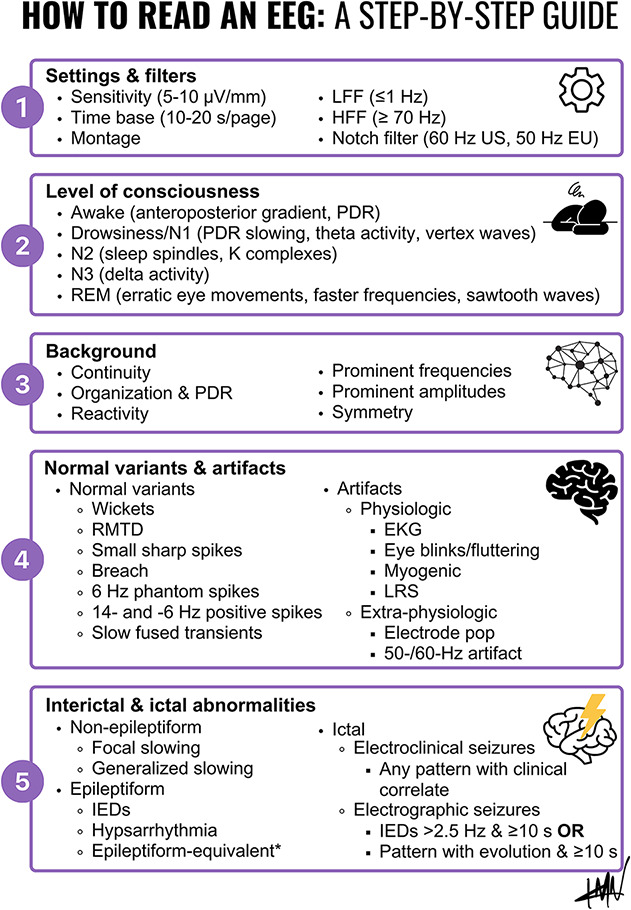
How to Read an EEG: A Step-by-Step Guide Educators should teach trainees on (1) technical aspects of EEG including settings and filters, (2) identifying level of consciousness based on video-EEG features, (3) systematically describing the EEG background activity, and accurately and reliably identifying (4) normal variants and artifacts, as well as (5) nonepileptiform, epileptiform interictal abnormalities, and ictal findings. *Epileptiform-equivalent: temporal intermittent rhythmic delta activity (TIRDA).^1^ EU = Europe; HFF = high frequency filter; IED = interictal epileptiform discharge; LFF = low-frequency filter; LRS = lateral rectus spikes; N2 = non-REM sleep, stage 2; N3 = non-REM sleep, stage 3; PDR = posterior dominant rhythm; REM = rapid eye movement; RMTD = rhythmic midtemporal theta burst of drowsiness; US = the United States.
